# Supplementary material for: Dynalign II: common secondary structure prediction for RNA homologs with domain insertions
Source: Nucleic Acids Res. 2014 Nov 21;42(22):13939–48. doi: 10.1093/nar/gku1172 (PMC4267632; doi:10.1093/nar/gku1172)
Supplement: SUPPLEMENTARY DATA [file supp_gku1172_nar-02021-z-2014-File012.zip › manual/Text/Thermodynamics.html]

RNAstructure Command Line Help: Specifying Thermodynamics


|  |  |  |
| --- | --- | --- |
|  | RNAstructure Command Line Help Specifying Thermodynamics | - Contents - Index |
| Specify the location of the thermodynamic parameters  RNAstructure uses a set of nearest neighbor thermodynamic parameters for predicting folding free energy changes of secondary structures. When an interface is invoked, these parameters are read from disk. The parameters come in the directory RNAstructure/data\_tables/. To indicate the location of this directory, an environment variable, DATAPATH, needs to be set to this location.  **In BASH, this is accomplished with:**  export DATAPATH=[directory in which RNAstructure resides]/RNAstructure/data\_tables/   **In CSH, this is accomplished with:**  setenv DATAPATH [directory in which RNAstructure resides]/RNAstructure/data\_tables/   **In DOS (Microsoft Windows), this can be done on the command line with:**  set DATAPATH=[driver letter on which RNAstructure resides]:\[directory in which RNAstructure resides]\RNAstructure\data\_tables   Note that, in Windows, environment variables can also be set using the graphical interface. On Windows XP, for example, this can be done from "System Properties" in the Control Panel. Click the "Advanced" tab and then click the ":Environment Variables" button. After specifying DATAPATH in the graphical interface, a reboot is required. | | |
| Visit The Mathews Lab RNAstructure Page for updates and latest information. | | |
